# Centiloid scaling for quantification of brain amyloid with [^18^F]flutemetamol using multiple processing methods

**DOI:** 10.1186/s13550-018-0456-7

**Published:** 2018-12-05

**Authors:** Mark R. Battle, Lovena Chedumbarum Pillay, Val J. Lowe, David Knopman, Bradley Kemp, Christopher C. Rowe, Vincent Doré, Victor L. Villemagne, Christopher J. Buckley

**Affiliations:** 1 Imaging Technology Group, GE Healthcare, The Grove Centre, White Lion Road, Amersham, Buckinghamshire UK; 2 Mayo Clinic, Rochester, MN USA; 3 Austin Health, Melbourne, Australia

## Abstract

**Introduction:**

A standardised method for quantifying β-amyloid PET tracers would allow comparison across different tracers and different sites. The development of the Centiloid scale has aimed to achieve this, applying a common scale to better aid the diagnosis and prognosis of Alzheimer’s disease (AD) and to monitor anti-amyloid therapeutic interventions. Here, we apply the Centiloid method to [^18^F]flutemetamol and [^11^C]PiB (PiB, Pittsburgh compound B) PET images and derive the scaling factor to express their binding in Centiloids.

**Methods:**

Paired PiB and [^18^F]flutemetamol scans for 74 subjects, including 24 young healthy controls (37 ± 5 years), were analysed using the standard Centiloid method. The same subjects were also analysed using PMOD- and FSL-based pipelines as well as SPM8. Test-retest analysis of 10 AD subjects was also performed with each pipeline.

**Results:**

The standard uptake value ratios (SUVR), determined using the standard SPM8 Centiloid process, showed a strong correlation between [^18^F]flutemetamol (Flute) and PiB binding (SUVR-Flute = 0.77 × SUVR-PiB + 0.22, *R*^2^ = 0.96). Application of the standard Centiloid process allowed the calculation of a direct conversion equation for SUVR-Flute to Centiloid units (CL) (CL = (121.42*SUVR-Flute) − 121.16). Analysis of the data via the two alternate Centiloid pipelines allowed us to derive standardised, SPM8-equivalent equations for both PMOD (CL = (115.24*SUVR-Flute) − 107.86) and FSL (CL = (120.32*SUVR-Flute) − 112.75) respectively. Test-retest analysis of 10 AD subjects showed an approximate 2% difference for each pipeline.

**Conclusions:**

[^18^F]flutemetamol data can now be expressed in Centiloid units, enhancing its utility in clinical and research applications for β-amyloid imaging. The standard Centiloid method also demonstrates that [^18^F]flutemetamol has favourable performance compared with PiB and other β-amyloid tracers. Test-retest difference averaged 2%, with no difference between image processing pipelines. Centiloid scaling is robust and can be implemented on a number of platforms.

## Background

Alzheimer’s disease (AD) is characterised by two key pathological findings, β-amyloid plaques and neurofibrillary tangles [1]. As possibly the earliest pathology, β-amyloid is a compelling therapeutic target [2]. The use of imaging biomarkers to visualise and measure the β-amyloid plaque load in individuals was introduced by Klunk et al., where a ^11^C-labelled Thioflavin-T-based molecule was developed to visualise amyloid plaque presence in vivo using PET imaging [3, 4]. Although [^11^C]PiB (PiB, Pittsburgh Compound-B) became the ‘Gold-Standard’ amyloid PET tracer for research studies, its use is limited by the short half-life (20 min) of ^11^C, requiring an on-site cyclotron when imaging with this tracer. This prompted the generation and clinical approval of ^18^F-labelled tracers (110 min half-life), allowing greater distribution and utilisation in PET centres [5]. Three tracers, [^18^F]florbetapir (Amyvid™), [^18^F]florbetaben (NeuraCeq™) and [^18^F]flutemetamol (Vizamyl™), are validated with post-mortem studies and have been approved by regulatory authorities [6–8]. Another tracer, [^18^F]NAV4694, has also been studied in a limited setting [9]. These tracers all show increased cortical retention in AD subjects. Each tracer has its own unique set of cortical and reference regions, methods for evaluation and positive PET cut-off points associated with quantitative use. In addition, differences in their dynamic range, kinetics and non-amyloid white matter binding all add up to make comparing data sets across studies, and across groups scanned with more than one of these tracers, complex.

Consequently, there is a need to standardise the methods for data collection and analysis to better aid cross-centre, multi-tracer utility. Standardised units would also allow better interpretation of longitudinal changes and improve how sites monitor disease progression and whether any potential therapeutic effects are observed.

The Centiloid Project was initiated to derive a standardised quantitative amyloid imaging measurement scale, based upon normalisation of data from the ^18^F-tracers to that of PiB. In this linear scale, young controls (≤ 45 years) have a mean of zero Centiloid units (CL) and typical mild to moderate AD patients score on average 100 CL [10]. The data set used to determine PiB Centiloids is freely available on the Global Alzheimer Association Interactive Network website (GAAIN; http://www.gaain.org), together with standardised cortical and whole cerebellum volume of interest (VOI) templates.

Standardised scaling, using Centiloids, has been recently reported for other amyloid tracers, [^18^F]florbetaben, [^18^F]NAV4694 and [^18^F]florbetapir, allowing comparison of these tracers with PiB [11–13].

This work reports the application of the Centiloid scaling methods to images obtained using [^18^F]flutemetamol (Vizamyl™, GE Healthcare). We also report the utility of alternate processing pipelines and assess the robustness and margin of error of the Centiloid analysis system through test-retest analysis on the standard and other processing systems.

## Materials and methods

### The Centiloid process

Klunk et al. provide details of the standard SPM8-based processing system (Statistical Parametric Mapping, version 8, Wellcome Trust Centre for Neuroimaging, http://www.fil.ion.ucl.ac.uk) and downloadable volumes of interest, PiB and T1 3D MRI image data sets and standard uptake values ratios (SUVR) results that should be obtained with this data if the method is executed correctly [10].

Klunk et al. also describe, in detail, the process to derive the transformation equation to convert other tracers SUVR into Centiloid units.

Using the downloaded GAAIN data sets, the local SPM8-based process pipeline should be first verified, by demonstration of a correlation with the published PiB data set, to ensure it meets the Centiloid method criteria of a slope between 0.98 and 1.02, an intercept between − 2 and 2 CL and an *R*^2^ > 0.98. The numerical relationship between the SUVRs obtained with the standard Centiloid method for [^18^F]flutemetamol and that using PiB in the same subjects are then determined. This will provide a linear equation that permits the [^18^F]flutemetamol SUVR (SUVR_Flute_) to be converted to PiB-equivalent SUVR (^PiB-Calc^SUVR) units. These ^PiB-Calc^SUVRs will then be converted into Centiloid units using the published data from Klunk et al. Finally, a linear equation that directly converts the SUVR_Flute_, obtained by the Centiloid method, into Centiloid units can be derived.

As per the Centiloid methods described by Klunk et al., to fully assess F-18 amyloid tracers requires scanning with both PiB and the F-18 tracer in the same subjects [10]. Here, the authors recommended that at minimum 25 subjects are included as a minimum cohort for tracer analysis. This group should comprise at least 10 young healthy controls (YHC), under the age of 45 years. This was based on the assumption that these YHC have no amyloid, allowing the variability in uptake (in the absence of amyloid) to be compared to PiB, by expression of the standard deviation of the F-18 tracer divided by the standard deviation of PiB in the same YHC (variance ratio). The analysis cohort should also comprise at least 15 subjects with a high likelihood of being amyloid positive, including subjects with typical AD and those with likely intermediate values of PiB retention (such as cognitively normal elderly subjects and/or aMCI subjects). Their rationale for this cohort was that a spread of data over the correlation range provides an increased validity to the correlation outcome measures compared with polar grouping of extreme low and high values only.

### [^18^F]flutemetamol subjects

Images analysed in this study were taken from two pivotal [^18^F]flutemetamol studies (ALZ201 and Mayo 103), where subjects had received both PiB and [^18^F]flutemetamol scans [14, 15]. Seventy-four subjects, 24 healthy young controls (YHC), comprising 10 males and 14 females aged under 45 years (37 ± 5 years, range 30 to 45 years), and 50 ‘Other’ subjects, comprising 20 males and 30 females, were evaluated in total. The ‘Other’ group consisted of 20 clinically diagnosed AD subjects (age 69 ± 10 years, range 60 to 82 years), 20 subjects with amnestic mild cognitive impairment (aMCI) (age 73 ± 7 years, range 57 to 83 years) and 10 older healthy controls (OHC), age 57 ± 11 years, range 47 to 75 years). The Centiloid scale calibration process requires a good spread of subjects across the range of amyloid burden, rather than a focus on the clinical diagnosis.

For test-retest analysis, a total of 10 subjects (6 males and 4 females) with confirmed AD (73 ± 6 years, range 56 to 81 years) were evaluated (5 subjects from Study GE067-017 and 5 from ALZ201) [16]. These subjects each received initial (test) [^18^F]flutemetamol scans, followed by a second (retest) scan within 2 weeks (10 ± 3 days, range 7 to 14 days).

### Validation of standard SPM8 Centiloid process

To validate our local standard SPM8 process pipeline, the ‘Level-1’ analysis (validation with PiB data), described by Klunk et al., was first replicated [10]. Briefly, the PiB data sets for 34 young control subjects (YC-0) and 45 subjects with confirmed Alzheimer’s disease (AD-100) were obtained from the GAAIN website. For processing with SPM8, PET and T1 3D MRI images were normalised to MNI-152 space, and the standard cortex (CTX) and whole cerebellum (WC) VOI templates were applied. SUVRs were obtained for both data set, and converted to Centiloids using the published equation for PiB (Klunk et al.). The derived results were compared to the standard results available on the GAAIN website.

### Derivation of standard conversion equation [^18^F]flutemetamol SUVR to Centiloids

Paired PiB and [^18^F]flutemetamol scans for the 74 study subjects, together with their corresponding T1 3D MRI images, were processed as per the standard SPM8 Centiloid method, using the whole cerebellum as the reference region to generate the SUVR values. The correlation between SUVR_PiB_ and SUVR_Flute_ determined the slope of (^*Flutemetamol*^*m*Flutemetamol) and intercept (^*Flutemetamol*^*b*Flutemetamol) values.

These values were then used to convert SUVR_Flute_ values into ^PiB-Calc^SUVR values, as per the calculation below:

1 $$ PiB-{\mathrm{Calc}}_{\mathrm{SUVR}}=\left({\mathrm{SUVR}}_{\mathrm{Flute}}{\hbox{-}}^{\mathrm{Flute}\mathrm{metamol}}b\right)/{}^{\mathrm{Flute}\mathrm{metamol}}m $$

The ^PiB-Calc^SUVR values were then converted to Centiloid values, using the published standard eq. CL = 100 × (^PiB-Calc^SUVR – ^PiB^SUVR_YC-0_) / (^PiB^SUVR_AD-100 -_^PiB^SUVR_YC-0_).

The equation to directly convert SUVR_Flute_ to Centiloid was derived by plotting each SUVR_Flute_ against the corresponding Centiloid value.

### Utility of alternate Centiloid processing pipelines

Once the SPM8 Centiloid scaling process was established, our aim was to evaluate the utility of two other commonly available image processing platforms, PMOD Image Quantification Software (www.pmod.com) and FSL (FMRIB Software Library, fsl.fmrib.ox.ac.uk), for application to [^18^F]flutemetamol images in transforming uptake on the Centiloid scale. For both pipelines, the GAAIN data were first replicated, following the previously described methods for the standard SPM8 process, to ensure validation of the Centiloid process on the individual pipelines. The paired images for the 74 study subjects were then evaluated on each process pipeline.

**Fig. 1:**
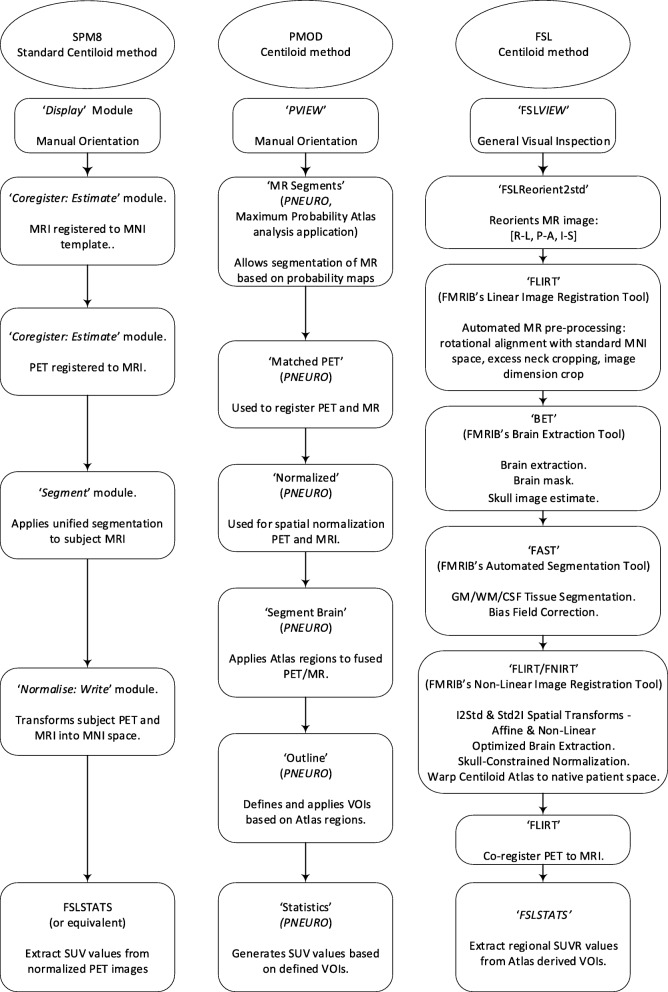
Process workflow for the standard SPM8 Centiloid methods, together with PMOD and FSL process methods

The process workflow for the standard SPM8 method is presented in Fig. 1, together with the process workflows for PMOD and FSL. Details of the process methods for each of the other pipelines are given below.

PMOD is a nuclear medicine image analysis system that can calculate PET image SUVRs using inbuilt or imported VOI masks. For processing the [^18^F]flutemetamol and PiB images in PMOD, the GAAIN VOIs (cortex, whole cerebellum, cerebellar grey, pons and whole cerebellum and brain stem) were imported and the derived atlas was applied to the images. Images were uploaded, reviewed and re-orientated using the PMOD’s PVIEW module. The PET and MR images were then co-registered and processed using the Maximum Probability Atlas analysis application in PMOD’s PNEURO module. The atlas was applied, and the output used to derive SUVRs for each region.

For Centiloid processing with FSL, a Python (v3.6) wrapper was built to provide a higher-level interface to the FSL (v5.0) processing routines. The Centiloid VOI images were reoriented with the anatomical co-ordinate system orientation of the MNI-152 template, before merging to form an atlas. Furthermore, critical to the robustness and accuracy of subsequent operations underlying brain extraction in FSL, PET-MR co-registration and back-projection of the Centiloid atlas to native patient space, both the PET and T1 MRI images underwent a series of pre-processing steps. The T1 MRI was rotated and cropped, making use of the FSL’s FLIRT (FMRIB’s Linear Image Registration Tool [17, 18]) in conjunction with the MNI-152 T1 whole head template (2 mm), a readily available translation-only 3D registration FLIRT schedule, and an MNI-152 spatially defined subcortical-biased weighted brain mask.

The forward rigid body transform was derived between a Gaussian-smoothed copy of the T1-w MRI and the MNI-152 whole-head template. This step was repeated using the translation-only 3D registration FLIRT schedule file, from which the inverse translation-only transform was estimated and combined with the forward rigid body transform resulting in a forward rotation-only transform; thus, a rotated MRI image in alignment with the MNI-152 template when applied to the original T1-w MRI was derived. Linear transformations were estimated and combined and applied in a final step to minimise errors introduced by interpolation.

Lastly, the inverse affine transform was derived between the MNI-152 whole-head template and rotated T1-w MRI, using the MNI-defined subcortical-biased weighted brain mask in the FLIRT registration. The resultant T1-registered MNI-152 whole head template was binarized and used to trim excess neck present in the MR image, yielding an appropriately reduced FOV MRI image for subsequent processing. The reoriented, rotated and cropped MRI image served as an input to the FSL’s BET (Brain Extraction Tool), generating an initial brain segmentation for use in the FSL’s FNIRT (FMRIB’s Non-Linear Image Registration Tool) [19, 20]. The FNIRT-derived non-linear coefficients were used to transform a MNI-152 spatially defined brain mask which was then applied to the processed T1-w MRI, refining the brain extraction. The non-linear coefficients were also used to warp the Centiloid atlas to native patient space.

Finally, the PET image was co-registered with the processed T1-w MRI via a rigid body transform with the FSL’s FLIRT, after which, together with the subjects T1-registered Centiloid atlas, regional SUVR measurements were extracted with the FSL’s FSLSTATS.

### Test-retest evaluation

Understanding the repeatability of the Centiloid process via test-retest has considerable value in that it provides an estimate of the likely variability of the process. This may offer a ‘delta’ in Centiloids which is the accuracy limit in comparing Centiloids from one subject’s [^18^F]flutemetamol image to another, or for monitoring individual efficacy of therapy.

Paired images for the 10 AD test-retest subjects were processed through all three Centiloid pipelines (SPM8, PMOD and FSL). SUVr values were obtained, and converted to Centiloids (as described previously). The difference between test and retest values were then calculated.

## Results

Validation of our local standard Centiloid (SPM8) process pipeline using the GAAIN data gave an excellent correlation, local SPM8 Centiloids = 1.00 × GAAIN Centiloids – 0.07 (*R*^2^ = 0.999), compared with the published data. This falls well within the minimum specified acceptance criteria defined by Klunk et al.

### Calibration of [^18^F]flutemetamol: standard SPM8 pipeline

**Fig. 2:**
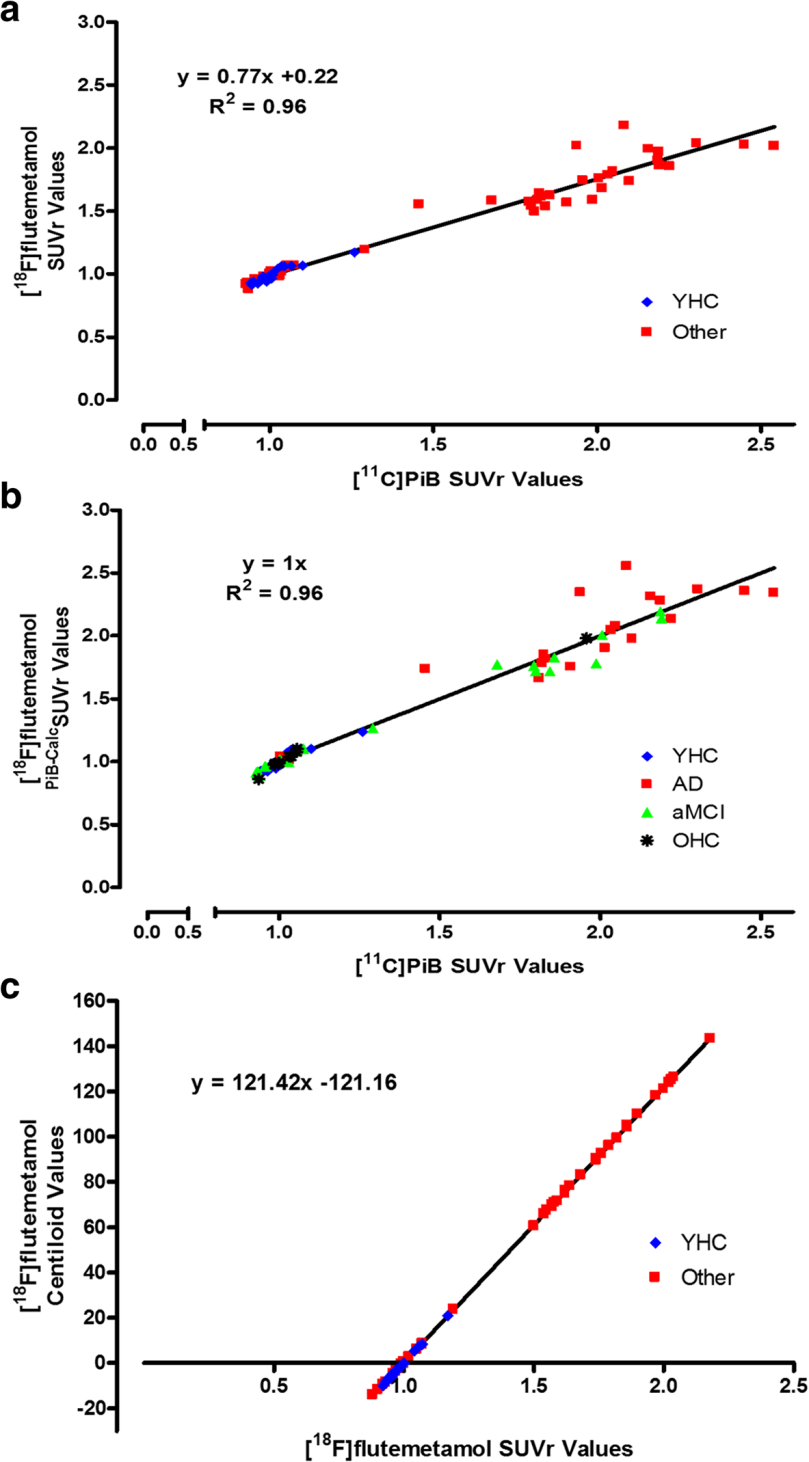
Correlation between SPM8-derived [^18^F]flutemetamol SUVR and [^11^C]PiB SUVR (**a**), PiB-Equivalent [^18^F]flutemetamol SUVR and PiB SUVR, further highlighting the wide spectrum of cognitive status comprising the ‘Other’ group (**b**) and [^18^F]flutemetamol Centiloids vs. [^18^F]flutemetamol SUVR’s (**c**). A total of 74 images (young healthy controls: *n* = 24, Other: *n* = 50) were processed

There was strong correlation between the PiB and [^18^F]flutemetamol SUVR values (*y =* 0.77*x +* 0.22), calculated using the Centiloid standard VOIs on the same-subjects, with *R*^2^ of 0.96 (Fig. 2a). This satisfied the Centiloid method relating to correlation between tracers (*R*^2^ > 0.70), confirming [^18^F]flutemetamol is a valid tracer for conversion through the Centiloid process. Figure 2b shows the correlation between PiB-equivalent [^18^F]flutemetamol and PiB SUVRs (*y = 1x, R*^*2*^ *= 0.96*), and further highlights the wider spectrum of cognitive status that comprise the ‘Other’ group. The correlation between [^18^F]flutemetamol Centiloids and SUVR values (SUVR_Flute_) allows us to derive the following conversion equation for SPM8 (Fig. 2c):

$$ \mathrm{CL}=\left(121.42\times {\mathrm{SUVR}}_{\mathrm{Flute}}\right)-121.16 $$

The mean (±SD) Centiloid values in the young healthy controls were − 1.0 ± 7.2 CL for [^18^F]flutemetamol and − 0.6 ± 6.1 CL for PiB, giving a variance ratio of 1.19.

### Assessment of alternate Centiloid processing pipelines

Validation of the PMOD and FSL image process pipelines was again confirmed using the GAAIN PiB data set. For PMOD, mean SUVRs of 0.98 ± 0.05 (range 0.91 to 1.08) and 2.07 ± 0.21 (range 1.61 to 2.42) were determined for the YC-0 and AD-100 groups respectively. PiB images processed using the FSL pipeline gave similar mean SUVR values for the YC-0 (1.00 ± 0.04, range 0.93 to 1.09) and AD-100 (2.08 ± 0.21, range 1.58 to 2.49) groups. These values were comparable to those generated using SPM8 (YC-0 1.01 ± 0.05, AD-100 2.09 ± 0.21) and were within 3% or less of the published GAAIN values (YC-0 1.01 ± 0.05, AD-100 2.08 ± 0.20). Correlation of the Centiloid values vs. the GAAIN published values gave a slope (*m*) of 0.999 and an intercept (*b*) of 0.040 (*R*^2^ = 0.998) for PMO, and 0.99 (*m*) and 0.05 (*b*) for FSL (*R*^2^ = 0.997). These slight deviations from the standard SPM8 Centiloid method (*x*) were corrected using the linear transforms of 0.997*x* + 0.146 for PMOD, and 0.997*x* + 0.159 for FSL pipelines.

**Fig. 3:**
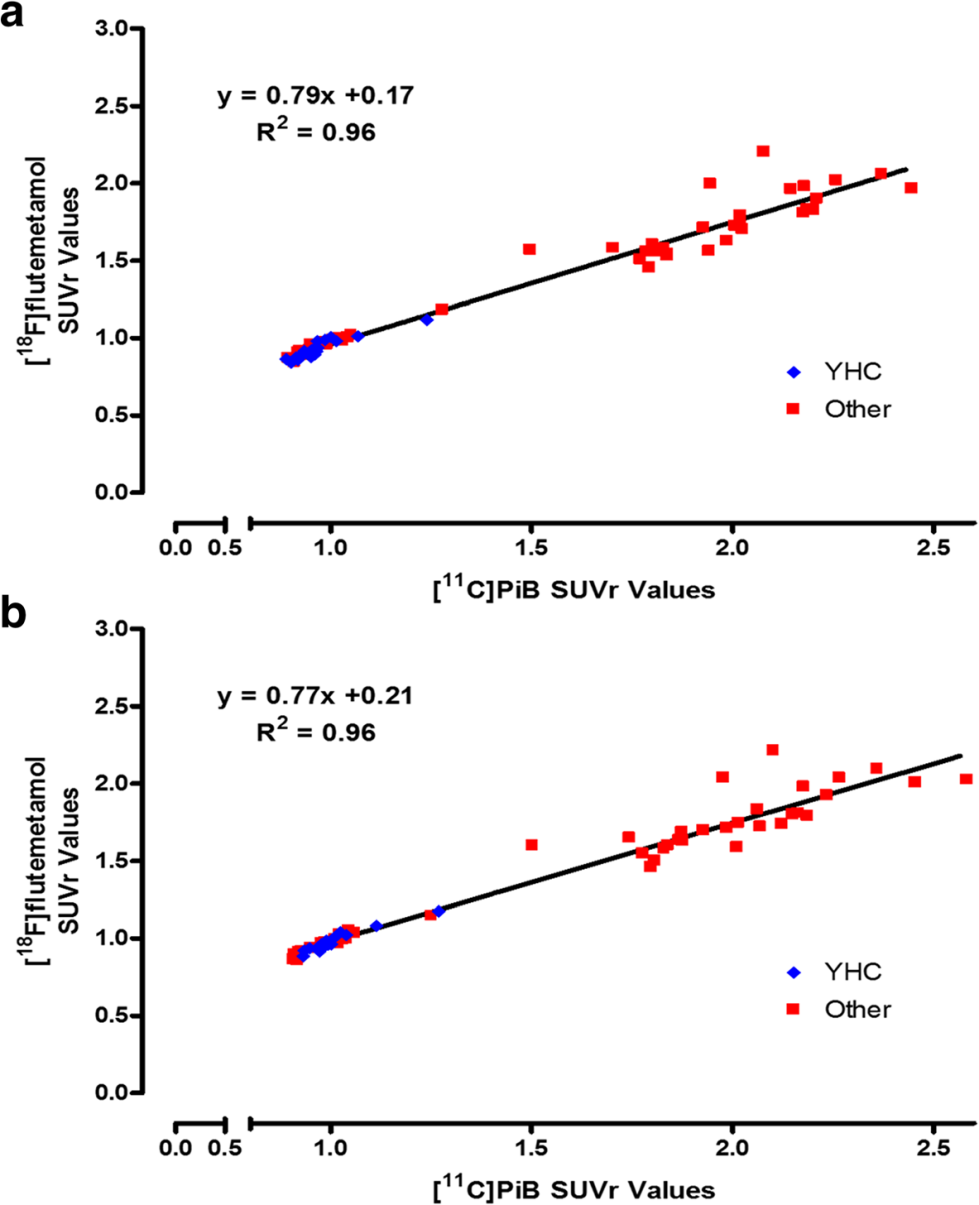
Correlation of PMOD-derived (**a**) and FSL-derived (**b**) [^18^F]flutemetamol SUVR vs. [^11^C]PiB

The paired PiB and [^18^F]flutemetamol images were then processed using PMOD and FSL. Analysis of the data for the site-acquired subjects showed there was good correlation between the SUVR_PiB_ and SUVR_Flute_ values in these subjects (Fig. 3). ^PiB-Calc^SUVR values were calculated, which were then converted to Centiloids.

The mean ^PiB-Calc^SUVR and Centiloid values for the YHC and ‘Other’ groups, together with the variance ratios, are presented in Table 1. The data show that the three process pipelines generate comparable data, with similar values for both ^PiB-Calc^SUVR and Centiloids.

**Table 1:** Mean (±SD) values for SUVR and Centiloid generated for paired PiB and [^18^F]flutemetamol using three different image process pipelines; SPM8, PMOD and FSL. Centiloid values for PMOD and FSL have not been corrected to ‘Standard’ (SPM8-equivalent) CL. For the variance ratio, SPM8-derived CL for young healthy controls were used as the gold standard

	[^11^C]PiB		[^18^F]flutemetamol*		Variance ratio^#^ (Centiloid)
	SUVR	Centiloid	^PiB-Calc^SUVR	Centiloid	
Young healthy controls (*n* = 24)					
SPM8	1.008 (0.065)	−0.6 (6.1)	1.003 (0.077)	−1.0 (7.2)	1.19
PMOD	0.967 (0.069)	−0.9 (6.3)	0.957 (0.079)	−1.8 (7.2)	1.14
FSL	1.003 (0.067)	2.4 (6.2)	1.000 (0.080)	2.2 (7.3)	1.18
Other (*n* = 50)					
SPM8	1.565 (0.528)	51.4 (49.2)	1.567 (0.539)	51.6 (50.3)	1.02
PMOD	1.549 (0.533)	52.4 (48.7)	1.554 (0.541)	52.8 (49.5)	1.02
FSL	1.575 (0.546)	54.8 (49.9)	1.577 (0.559)	54.9 (51.1)	1.02

*PiB-Equivalent SUVR [^18^F]flutemetamol values. #Centiloid SD [^18^F]flutemetamol/Centiloid SD PiB

After testing for normality, the nonparametric Kruskal-Wallis test (Minitab v12.3) was conducted to examine the differences in SUVR and Centiloid values generated for each data set by each of the three process pipelines. No significant differences were found for either the SUVR values (*p* = 0.46) or the Centiloid values (*p* = 0.38) among the three process methods (SPM8, PMOD and FSL).

**Fig. 4:**
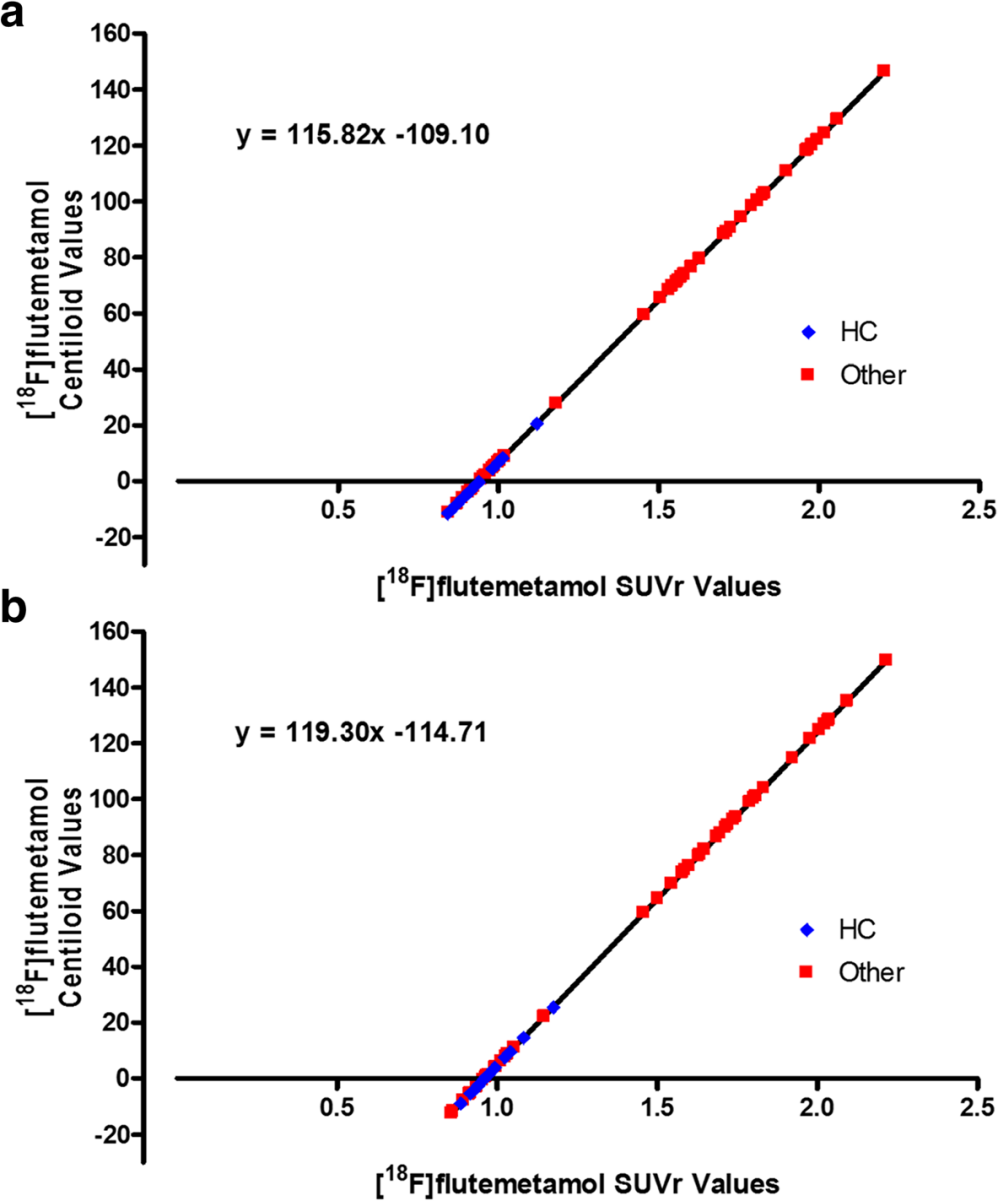
The correlation between the [^18^F]flutemetamol SUVRs and corresponding Centiloids for PMOD-processed (**a**) and FSL-processed (**b**) paired [^11^C]PiB and [^18^F]flutemetamol images

The correlation between the [^18^F]flutemetamol SUVRs and Centiloids was obtained for PMOD and FSL (Fig. 4).

The PMOD and FSL-generated Centiloids were then transformed to ‘Standard’ SPM8-equivalent Centiloids through correlation of the pipeline-derived Centiloids with the SPM8-derived Centiloids (*x*), resulting in the following equations for PMOD and FSL.

$$ \mathrm{PMOD}:\mathrm{CL}=\left(115.24\times {\mathrm{SUVR}}_{\mathrm{Flute}}\right)-107.86 $$

$$ \mathrm{FSL}:\mathrm{CL}=\left(120.32\times {\mathrm{SUVR}}_{\mathrm{Flute}}\right)-112.75 $$

### Test-retest

To determine the robustness of the Centiloid system and to assess the margin of error obtained with it, test-retest data from 10 AD subjects was analysed using each image process pipeline. For each process pipeline, average SUVR_Flute_ values of approximately 1.8 SUVR units were observed for both test and retest (Table 2). No significant differences were observed between the test and retest values for any subject, when processed on each platform (*t* test, *p* > 0.05).

**Table 2:** Mean (±SD) SUVR, SUVR-1 and Centiloid values generated for [^18^F]flutemetamol test-retest subject. Images from 10 AD subjects, average age 73 ± 6 years, were analysed using three Centiloid process pipelines: SPM8, PMOD and FSL

	SPM8			PMOD			FSL		
	SUVR	SUVR-1	Centiloid	SUVR	SUVR-1	Centiloid	SUVR	SUVR-1	Centiloid
Test	1.80 (0.18)	0.80 (0.18)	96.4 (21.2)	1.80 (0.18)	0.80 (0.18)	98.9 (20.5)	1.83 (0.21)	0.83 (0.21)	102.8 (25.6)
Retest	1.81 (0.18)	0.81 (0.18)	97.7 (21.3)	1.81 (0.18)	0.81 (0.18)	100.5 (21.1)	1.83 (0.21)	0.83 (0.21)	103.3 (25.3)
% Difference	0.6 (1.7)	1.6 (4.1)	1.6 (4.0)	0.8 (1.6)	1.8 (3.7)	1.7 (3.4)	0.3 (1.7)	0.8 (4.3)	0.8 (4.1)

The percentage difference between test and retest values on each pipeline was approximately 1% for SUVR. When calculated as SUVR-1, the test-retest difference was approximately 2% for each pipeline. The conversion of SUVR to Centiloid scaling again reflects these differences, with approximately 2% difference in test-retest Centiloid values for each pipeline.

Comparison of the process pipelines also found no statistically significant differences in SUVR values for test (*p* = 0.76) or retest (*p* = 0.86) or Centiloid test (*p* = 0.58) and retest (*p* = 0.60) values between each process pipeline: SPM8, PMOD or FSL (Kruskal-Wallis test). The average percentage difference in Centiloid values between SPM8 and PMOD was 2.9 ± 3.0% (test) and 3.0 ± 3.9% (retest). There was a slight difference between FSL and the other pipelines, with 6.2 ± 9.6% (SPM8 vs FSL) and 3.2 ± 9.6% (PMOD vs FSL) for the test data. For the retest data, the difference was 5.2 ± 7.5% (SPM8 vs FSL) and 2.2 ± 7.5% (PMOD vs FSL). Much of this variation was accounted for by a single subject, where issues (segmentation of the cerebellum VOI extended beyond the brain tissue into the CSF, plus this subject had a high degree of atrophy in the cortical regions) with the reference region in FSL led to a greater SUVR, and hence Centiloid, value for that subject in FSL vs the other pipelines. With that subject excluded the average differences fell was more comparable with that of PMOD and SPM8, with 3.5 ± 4.9% and 3.4 ± 5.3% (FSL vs SPM8) and 0.5 ± 4.1% and 0.1 ± 3.9% (FSL vs PMOD) for test and retest data respectively.

## Discussion

A standardised, robust method of quantification is key to utilising amyloid PET in disease prediction, diagnosis and progression. The methods described by Klunk et al. have provided the framework to allow sites to compare multiple tracers using a single scale, Centiloids. Essential to this method of analysis is the accurate establishment and calibration of the processing pipeline. Here, we have described and defined the conversion of [^18^F]flutemetamol SUVR derived using the standard Centiloid methods (SPM8) to Centiloid units. SPM8 was the recommended image processing platform described in the methods of Klunk et al. and was selected by us when replicating the process pipeline. More recent versions of SPM have been introduced since, with the use of SPM12 reported by other groups [21]. However, we chose to use SPM8 to ensure we followed the process as accurately as possible. We also investigated the utility of two alternate image processing software platforms (PMOD and FSL) to replicate the Centiloid process and generate [^18^F]flutemetamol results. These platforms were selected as they were available and routinely used in-house.

Whole cerebellum was selected as the reference region for use with Centiloid processing. This differs from the preferred reference region, cerebellar cortex, recommended for [^18^F]flutemetamol. Again, whole cerebellum was used in this study in order to ensure the accurate replication of the Centiloid process. The use of alternate reference regions may be investigated in future work.

The data show that [^18^F]flutemetamol is a suitable tracer for Centiloid conversion, using the standard SPM8 process. There was strong correlation between [^18^F]flutemetamol and PiB, indicating that the tracers show similar uptake and kinetics. This was also apparent from the low variance values observed for the YHC subjects. When compared with other tracers, [^18^F]flutemetamol performed favourably when converted to Centiloids, with a better variance ratio (1.19) than that reported for [^18^F]florbetaben (1.96) and [^18^F]florbetapir (4.62) [11, 13]. This would be expected given the similar chemical structures of PiB and [^18^F]flutemetamol.

The utility of PMOD and FSL to process [^18^F]flutemetamol images and derive SUVR values, for conversion to Centiloid, was also assessed in this study. Both PMOD and FSL performed well, producing data that was comparable to the standard SPM8 methods. Validation of each pipeline against the GAAIN data again gave excellent correlation when compared with the published data, with both pipelines fulfilling the required acceptance criteria. The equations derived for each method were then transformed to ‘Standard’ SPM8-equivalent Centiloids, allowing a true comparison of values. This shows that Centiloid scaling is robust and can be implemented on a number of platforms, not just the initially recommended SPM8 version. Furthermore, this work provides a straightforward framework for implementation and validation of Centiloid scaling using any other processing pipeline.

Further, the test-retest difference has been assessed and produced an average of approximately of 2% or less between test and retest Centiloid values on each pipeline. The different image processing pipelines did not produce significantly different results for the test-retest data, with an average of ≤ 2% difference between these pipelines: SPM8, PMOD and FSL.

Previously published data for a small cohort of AD subjects found the SUVR test-retest variability in a composite cortical region averaged 1.5% (range 0.9 to 2.4%, *n* = 5), comparable with our findings [14]. However, SUVR scales have a measurement offset which makes SUVR percentage differences in test-retest look more favourable than those for binding potential (BP), and differences in SUVR-1 test-retest values are a better comparison as BP can be approximated by SUVR-1 [22, 23]. Importantly, the methodology of creating Centiloid values has an in-built subtraction of average healthy control SUVRs to zero, therefore providing a more robust measure than direct SUVR percentage differences that is similar to that for SUVR-1. The low difference in test-retest Centiloid results is comparable with the difference in estimated binding potential related measurements (SUVR-1).

This low difference in the test-retest data, and the favourable variance in young healthy controls means that [^18^F]flutemetamol Centiloid are suitable for comparing images, or monitoring brain amyloid levels in therapeutic clinical trials, with a high degree of sensitivity to small changes.

This work will allow us to analyse and compare data from various studies, a key future aim of the Centiloid project. The conversion equation allows us to apply Centiloid scaling to subjects for which [^18^F]flutemetamol/MRI scans but no PiB data are available for analysis.

The use of different scanners in this study was not taken in to account when analysing the data. It would be interesting to further investigate this to fully understand any variations and help interpret the results.

Further, a recent study by Bourgeat et al. looked at the utility of an alternate image processing platform, CapAIBL [24]. CapAIBL utilises a PET-only approach, overcoming the requirement for a corresponding MR image, and is particularly useful where MR imaging is not possible. The authors reported similar results for the conversion of [^18^F]flutemetamol through this pipeline. The application of such PET-only quantification methods could lead to a readily adopted clinical quantification method as images could be processed directly from the PET scanner. To that end, further work to investigate the utility of a PET-only method based on CortexID (AW Workstation, GE Healthcare), a dedicated platform for reviewing [^18^F]flutemetamol images is on-going.

## Conclusion

[^18^F]flutemetamol data can now be expressed in Centiloid units, enhancing its utility in both clinical and research applications for β-amyloid imaging. Standardised quantification can provide supplementary information to compliment visual assessment, especially in equivocal cases, and also provide a means to assess patients longitudinally. The standard Centiloid method also demonstrates that [^18^F]flutemetamol has favourable performance compared with PiB and other β-amyloid tracers. Test-retest difference averaged 2%, with no difference between image processing pipelines. Centiloid scaling is robust and can be implemented on a number of platforms.

## Abbreviations

AD: Alzheimer’s disease
aMCI: Amnestic mild cognitive impairment
CL: Centiloid units
CTX: Standard cortex
Flute: [^18^F]Flutemetamol
FSL: FMRIB Software Library
GAAIN: Global Alzheimer Association Interactive Network
OHC: Older healthy controls
PiB: Pittsburgh compound B
SPM8: Statistical Parametric Mapping, version 8
SUVR: Standard uptake value ratios
VOI: Volume of interest
WC: Whole cerebellum
YHC: Young healthy controls
